# MicroRNA profiling of the intestinal tissue of Kazakh sheep after experimental *Echinococcus granulosus* infection, using a high-throughput approach

**DOI:** 10.1051/parasite/2016023

**Published:** 2016-05-27

**Authors:** Song Jiang, Xin Li, Xuhai Wang, Qian Ban, Wenqiao Hui, Bin Jia

**Affiliations:** 1 College of Animal Science and Technology, Shihezi University Road Beisi Shihezi 832003 Xinjiang PR China; 2 Institute of Animal Husbandry and Veterinary Medicine, Anhui Academy of Agriculture Sciences Road Nongkenan Hefei 230031 Anhui PR China; 3 Center for Stem Cell and Translational Medicine, School of Life Sciences, Anhui University Road Jiulong Hefei 230000 Anhui PR China

**Keywords:** microRNA, Cystic Echinococcosis, Sheep, MHC haplotype

## Abstract

Cystic echinococcosis (CE), caused by infection with the larval stage of the cestode *Echinococcus granulosus*, is a chronic zoonosis, to which sheep are highly susceptible. Previously, we found that Kazakh sheep with different MHC haplotypes differed in CE infection. Sheep with haplotype MHC*Mva*Ibc-*Sac*IIab-*Hin*1Iab were resistant to CE infection, while their counterparts without this haplotype were not. MicroRNAs (miRNAs), a class of small non-coding RNAs, are key regulators of gene expression at the post-transcriptional level and play essential roles in fundamental biological processes such as development and metabolism. To identify microRNA controlling resistance to CE in the early stage of infection, microRNA profiling was conducted in the intestinal tissue of sheep with resistant and non-resistant MHC haplotypes after peroral infection with *E. granulosus* eggs. A total of 351 known and 186 novel miRNAs were detected in the resistant group, against 353 known and 129 novel miRNAs in the non-resistant group. Among these miRNAs, 83 known miRNAs were significantly differentially expressed, including 75 up-regulated and 8 down-regulated miRNAs. Among these known microRNAs, miR-21-3p, miR-542-5p, miR-671, miR-134-5p, miR-26b, and miR-27a showed a significantly higher expression in CE-resistant sheep compared to the CE-non-resistant library, with the FC > 3. Functional analysis showed that they were NF-kB pathway-responsive miRNAs, which are involved in the inflammation process. The results suggest that these microRNAs may play important roles in the response of intestinal tissue to *E. granulosus*.

## Abstract (fr)

L’échinococcose kystique (EK), causée par infection par le stade larvaire du cestode *Echinococcus granulosus*, est une zoonose chronique, à laquelle les moutons sont particulièrement sensibles. Auparavant, nous avons constaté que les moutons Kazakh avec différents haplotypes de CMH différaient dans l’infection par EK. Les moutons avec l’haplotype MHC MvaIbc-SacIIab-Hin1Iab étaient résistants à l’infection par EK, tandis que leurs homologues sans cet haplotype ne l’étaient pas. Les microARN (miARN), une classe de petits ARN non-codants, sont des régulateurs-clés de l’expression des gènes au niveau post-transcriptionnel et jouent des rôles essentiels dans les processus biologiques fondamentaux tels que le développement et le métabolisme. Pour identifier les microARN contrôlant la résistance à EK dans le stade précoce de l’infection, un profilage des microARN a été réalisé dans le tissu de l’intestin des moutons d’haplotypes MHC résistants et non-résistants, après infection perorale par des œufs d’*E. granulosus*. Un total de 351 miARN connus et 186 miARN nouveaux ont été détectés dans le groupe résistant, contre 353 connus et 129 nouveaux dans le groupe non-résistant. Parmi ces miARN, 83 des connus étaient exprimés de manière significativement différente, dont 75 régulés à la hausse et 8 régulés à la baisse. Parmi ces microARN connus, miR-21-3p, miR-542-5p, miR-671, miR-134-5p, miR-26b et miR-27a ont montré une expression significativement plus élevée chez les moutons résistants à EK comparés à la bibliothèque des non-résistants, avec FC > 3. Une analyse fonctionnelle a montré que ce sont des miARN de la voie NF-kB, qui interviennent dans le processus d’inflammation. Les résultats suggèrent que ces microARN peuvent jouer des rôles importants dans la réponse du tissu intestinal à *E. granulosus*.

## Introduction

Cystic echinococcosis (CE) is a chronic parasitic zoonosis caused by infection with the larval stage of the cestode *Echinococcus granulosus*, resulting in the development of cysts in human and domestic animals. In domestic animals, especially in sheep, which appear to be highly susceptible to infection, CE causes considerable health problems and thereby significantly affects the income of herdsmen.

It has been demonstrated that the pathogenesis of CE is strongly influenced by genetics, and certain MHC polymorphisms are associated with individual susceptibility in humans [1, 2]. In domestic animals, we have carried out several studies to investigate the relationship between *MHC*-*DRB*1/DQB1 gene polymorphism and genetic resistance or susceptibility to CE in Chinese Merino, Duolang, and Kazakh sheep [9, 16, 22, 23]. Surprisingly, we have identified genetic markers (MHC MvaIbc-SacIIab-Hin1Iab haplotype) for CE resistance in Kazakh sheep. The rate of *E. granulosus* infection in the internal organs of Kazakh sheep with this haplotype is confirmed to be significantly lower than in sheep without this haplotype, when exposed to the same number of parasites [22]. CE-resistant sheep therefore have an increased genetic capability to respond to and subsequently reject parasites when challenged.

Subsequently, we made several attempts to understand the mechanism of resistance to CE in sheep with this resistance haplotype. Li et al. found that Th1 cytokines were predominant in sheep with the CE resistance haplotype, which could induce protective immunity, while in sheep without this haplotype, Th2 cytokines were predominant, which could induce susceptibility to CE infection [23]. Hui et al. [17] found that the differences in CE resistance in sheep with different MHC haplotypes could be partially mediated by Th1-type cytokines and IgE, in the early stage of *E. granulosus* infection. It is therefore concluded that the intestinal tissue of sheep may have a certain function in parasite invasion. It has been demonstrated in mice that intestinal mucosal immunity, as a first line of defense, could be involved in stopping larval growth of *E. granulosus* at the very first stages [25]. Furthermore, using suppression subtractive hybridization (SSH) methods to detect different expressed sequence tags (ESTs) in the intestinal tissue of sheep infected with *E. granulosus*, Du et al. [9] found that other immune parameters such as IgM and non-immune factors are involved in induction of these enhanced levels of resistance. However, there remain many unanswered questions about the precise molecular and cellular mechanisms underlying the difference in CE resistance in sheep.

MicroRNAs (miRNAs) are small 20–22 nucleotide (nt) non-coding RNAs, which play a variety of roles in diverse biological processes at the post-transcriptional regulatory level in tissues during animal development [11]. Protozoan miRNAs and components of their silencing machinery possess features different from other eukaryotes, which can provide some clues on the evolution of the RNA-induced silencing machinery. miRNA functions possibly associate with neoblast biology, development, physiology, infection, and immunity of parasites. So far, a number of miRNAs have been identified in parasites, including *Plasmodium* [7], *Echinococcus* [3, 8, 24], schistosomula [14, 27], fluke *Fasciolopsis buski* [5], etc.

Since parasite infection can alter host miRNA expression, it can favor both parasite clearance and infection. miRNA pathways are, thus, a potential target for the therapeutic control of parasitic diseases. However, there is little literature on microRNA profiles in the intestinal tissue after echinococcosis infection, which is the first organ that the parasite invades. It is, therefore, necessary to conduct genome-wide characterization of microRNA profiles involved in resistance to CE infection in sheep, especially in sheep with different MHC haplotypes. In this study, to identify microRNA controlling resistance to CE in the early stage of infection, microRNA profiling was conducted in the intestinal tissue of sheep with resistant (R) and non-resistant (NR) MHC haplotypes after peroral infection with *E. granulosus* eggs.

## Materials and methods

### Animals and experimental design

The Animal Care and Use Committee of Shihezi University approved all procedures and experiments. Healthy 2-year-old ewes with CE-resistant [*MHCMva*Ibc-*Sac*IIab-*Hin*1Iab(R)] and CE-non-resistant [*MHCMva*Ibc-*Sac*IIab-*Hin*1Iab(NR)] haplotypes were selected, according to our previous method [21], and were raised in the farm of Shihezi University. All of the animals were raised under the same conditions of free access to water and food in natural lighting.

These animals were divided into two groups according to their MHC haplotype: three sheep with the CE-resistant (R) haplotype were referred to as group R, while the other three sheep with the CE-non-resistant (NR) haplotype constituted group NR. Sheep were screened for absence of hydatid cysts and were healthy prior to the experiment. According to the previous approach, the sheep were orally infected with *E. granulosus* eggs [18]. All the animals were sacrificed at 8 hours post-infection. Immediately afterwards, the intestinal tissue was removed and approximately 100 mm^3^-sized intestinal tissue blocks were collected and frozen immediately in liquid nitrogen prior to long-term storage at −80 °C until RNA extraction.

### Small-RNA library preparation and Illumina sequencing

The intestinal tissue was removed from the liquid nitrogen and the total RNA was extracted using TRIZOL (Invitrogen, USA), according to the manufacturer’s protocol. The quality and quantity of RNA samples were checked by an Agilent 2100 Bioanalyzer. The RNA from three sheep in each group was pooled to generate the two libraries. RNA length ranging from 18 to 30 nt was isolated and purified. Proprietary (Solexa) adaptors were then added to the 3′ and 5′-termini of sRNAs and subsequently used for cDNA synthesis. These ligation products were amplified by reverse transcription PCR using an RT-PCR kit (Takara, China). The produced libraries were sequenced using a Solexa sequencer at the Beijing Genomics Institute (BGI)-Shenzhen, China, according to the manufacturer’s instructions. Image analysis and base calling were performed with the Illumina built-in SCS2.8/RTA1.8 software.

### Read mapping and annotation

To obtain clean and unique small-RNA reads, the fastx_toolkit (v0.0.13.2, downloadable at http://hannonlab.cshl.edu/fastx_toolkit/) was used to filter off low-quality contamination reads. The clean reads were screened against and mapped to the ovine genome assembly (Oar_v3.1, released September 20, 2012) (ftp://ftp.ncbi.nlm.nih.gov/genomes/ASSEMBLY_REPORTS/All/GCF_000298735.1.assembly.txt), using the SOAP (Supplemental Offer and Acceptance Program). Afterwards, we used the BLAST tool on the clean reads against the Rfam database and GenBank to annotate and remove reads for mRNA, tRNA, rRNA, snoRNA, and snRNA. Then, the remaining sequences were searched against the mature miRNAs of animals in miRBase to identify known conserved miRNA homologs in sheep. If small-RNA mature and precursor sequences were perfectly matched to known ovine miRNAs in the miRBase, they were considered conserved miRNAs. Sequences that were identical to or related to the reference mature miRNAs were annotated as miRNA candidates.

### Identification of conserved and novel miRNAs

In order to discover potential new miRNAs, the surrounding 300 bases (150 upstream and downstream) flanking each unique miRNA candidate sequence were obtained and their folding secondary structures were determined using the miReap program.

After prediction, the resulting potential miRNA loci were examined carefully based on the distribution and numbers of small RNAs on the entire precursor regions. Those sequences residing in the stem region of the stem-loop structure and ranging between 20 and 22 nt with free energy hybridization lower than −20 kcal/mol were considered as potential new miRNAs.

### Determination and target prediction of differentially expressed miRNAs

A list of the differentially expressed genes with a 2-fold difference was generated and controlled with a false discovery rate (FDR) at 0.05 in an experiment. To predict potential miRNA target genes, the differentially expressed miRNAs were analyzed using the MIREAP and TargetScan software to predict potential.

### Statistical analysis

All of the data are presented as the means ± SD. A Student’s *t*-test was performed and *P* < 0.05 was considered statistically significant, when group comparisons were made.

## Results

### Summary of the sequence reads

**Figure 1.**
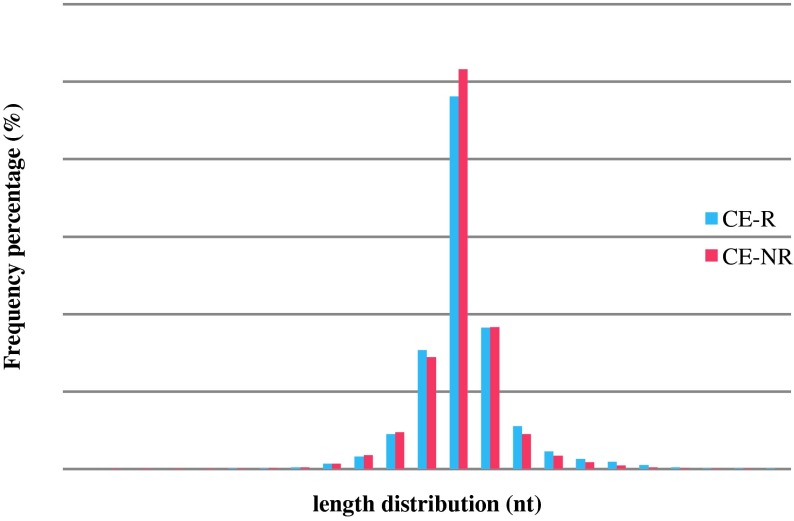
The size distribution of the small RNAs in CE-R and CE-NR libraries. (CE-R: sheep with CE-resistant (R) haplotype; CE-NR: sheep with CE-non-resistant (NR) haplotype).

In this study, miRNA from the intestinal tissue of CE-R and CE-NR sheep was sequenced. Firstly, we generated a small-RNA library from sheep with different haplotypes. Illumina sequencing of the purified cDNA libraries generated 28,842,662 total raw reads: 13,800,608 (13.8 million) from the CE-R library and 15,042,054 (15.0 million) from the CE-NR library (Table 1). To assess the efficiency of sequence quality, all reads were annotated and classified by aligning against the miRBase 20.0 database, Rfam database, Repbase database, ovine genome database, and the mRNA database. Of these reads, 72.85% (5.80/7.78 million reads, R) and 76.13% (2.11/4.19 million reads, NR) were mapped to the ovine genome Oar_v3.1. Figure 1 illustrates the summary of data cleaning and length distribution of tags in the two groups. Table 2 displays the information on distribution of small RNA among different groups.

**Table 1.** Summary of reads from raw data to cleaned sequences for small RNAs in CE-R versus CE-NR in sheep.

Item	CE-R group library		CE-NR group library	
	Total (%)	Unique (%)	Total (%)	Unique (%)
Total reads	13,587,724 (100%)	748,307 (100%)	14,646,574 (100%)	571,606 (100%)
Exon-antisense	6775 (0.05%)	2962 (0.4%)	5063 (0.03%)	1738 (0.3%)
Exon-sense	133,866.00 (0.99%)	111,877.00 (14.95%)	106,838.00 (0.73%)	90,046.00 (15.75%)
intron_antisense	312,754.00 (2.3%)	19,491.00 (2.6%)	219,448.00 (1.5%)	13,581.00 (2.38%)
Intro-sense	329,744.00 (2.43%)	55,295 (7.39%)	297,407.00 (2.03%)	35,607.00 (6.23%)
miRNA	8,606,510.00 (63.34%)	4269.00 (0.57%)	10,035,170.00 (68.52%)	3779.00 (0.66%)
rRNA	439,523.00 (3.23%)	34,101.00 (4.56%)	419,864.00 (2.87%)	35,424.00 (6.20%)
Repeat	130,348.00 (0.96%)	64,087.00 (8.56%)	70,553.00 (0.48%)	31,278.00 (5.47%)
snRNA	18,297.00 (0.13%)	4636.00 (0.62%)	13,307.00 (0.09%)	3567.00 (0.62%)
snoRNA	20,684.00 (0.15%)	3995.00 (0.53%)	12,220.00 (0.08%)	2889.00 (0.51%)
tRNA	139,213.00 (1.02%)	17,943.00 (2.40%)	111,653.00 (0.76%)	14,785.00 (2.59%)
unann	3,449,510.00 (25.39%)	429,651.00 (57.42%)	3,355,111.00 (22.91%)	338,912.00 (59.29%)

**Table 2.** Summary of known miRNAs in each group.

Item	Known miRNA in miRbase	CE-R	CE-NR
miRNA	672	351	353
miRNA-5p	132	78	74
miRNA-3p	132	81	80
miRNA precursors	903	513	517
Unique precursors matched to miRNA precursors	−	4349	3875
Total precursors matched to miRNA precursors	−	8,608,160	10,037,028

### Identification of known sheep miRNAs and prediction of potential novel miRNAs

To identify known miRNAs in sheep intestines, the data set was compared with the known mammalian miRNAs (miRNA precursors and mature miRNAs) in miRBase 20.0 (ftp://mirbase.org/pub/mirbase/CURRENT/). Sequences with a perfect match or one mismatch were retained in the alignment. We identified 351 known miRNAs in the CE-R library and 353 in the CE-NR library (Table 2).

In the present study, we identified three miRNA groups: (a) miRNAs previously described in sheep, (b) miRNAs described in species other than sheep, and (c) new miRNAs without any known annotations. In total, we identified 186 novel miRNAs in the R library and 129 in the NR library.

### Differential expression of miRNAs in the CE-R and CE-NR groups

Pairwise comparisons revealed significant differential expression of miRNAs between the two groups. Although some of their expression quantities were equivalent, some miRNAs were expressed differently. We focused on miRNAs meeting our designated criteria of *P*-values < 0.05 and |log2 (fold change)| > 1, and a total of 83 (75 up and 8 down) differentially expressed miRNAs were selected from the CE-R and CE-NR libraries (Supplementary Table). The normalized count data are given in Supplementary Table, showing the statistically significant differentially expressed miRNAs.

### Target gene predictions for differentially expressed miRNAs

A total of 22876 annotated mRNA transcripts were predicted as putative target genes for the 83 differentially expressed miRNAs.

## Discussion

As technological advancements have resulted in increased discovery of new miRNAs, in the present study, using RNA deep sequencing technology, we found a large number of new miRNAs in studied Kazakh sheep. These new miRNAs were all confirmed as having a hairpin secondary structure in their predicted precursor sequences.

The alterations of host miRNA expression reflect the roles of host-derived miRNAs in parasite infection [20]. It is estimated that up to 30% of genes might be regulated by miRNAs. In a separate study, we demonstrated predominant up-regulation of immune factors in the early stage of infection of CE-resistance sheep. However, the regulatory mechanisms for up-regulation were not fully elucidated. The current study has identified 83 differentially expressed miRNAs in the two CE infection sheep groups (resistance and non-resistance), of which, 75 were up-regulated and 8 were down-regulated in the resistance group. The target prediction and integration of these differentially expressed miRNAs with mRNA-seq data generated in our separate study helped to identify the most significant miRNAs and the potential target genes that could be responsible for the resistance ability to CE infection.

Among the known differential expression of miRNAs, miR-21-3p, miR-542-5p, miR-671, miR-134-5p, and miR-26b, miR-27a showed a much higher expression in CE-R sheep compared to the CE-NR library, in terms of normalized read counts (fold changes were 3.37, 3.5, 6.88, 5.25, 3.75, and 3.2, respectively). These miRNAs were already known to be crucially involved in immunity to disease in humans.

In particular, microRNA-21 (miR-21) was found to be up-regulated in cervical cancer tissues [13] Accumulating evidence for differential expression of miR-21 in cervical cancer suggests that it might play a crucial role in tumor biology [15]. It has been confirmed that miR-21 was transactivated through promoter binding of NFKB-P65. In parasite disease, it had been found that inhibition of miR-21 resulted in increased *Cryptosporidium parvum* burden [28]. Conversely, negative feedback regulation of TLR4/NF-kB signaling may be reached by miR-21 induction after *C. parvum* infection, as miR-21 targets PDCD4, a proinflammatory protein that promotes activation of NF-kB and suppresses interleukin 10. In the present study, significant overexpression of miR-21 was obtained in CE-resistant sheep, which suggested that it might be a biomarker associated with CE resistance.

Moreover, we also found that microRNA-542-5p was significantly up-regulated in CE-resistant sheep, when compared with their counterparts with non-resistance. MicroRNA-542-5p has been proven to be a novel tumor suppressor in neuroblastoma; overexpression of microRNA-542-5p decreases the invasive potential of neuroblastoma cell lines *in vitro* [19]. In our study, we find that microRNA-542-5P is highly expressed in sheep with CE resistance, suggesting that it may be related to defense against the parasite.

MiR-134-5p is related to the functioning of B cells. It has been reported that miR-134-5p expression is significantly up-regulated in infectious mononucleosis caused by primary Epstein-Barr virus infection in children [12]. MicroRNA-26b modulates the NF-κB pathway in alveolar macrophages by regulating PTEN [26].

MiR-27a is a member of the miR-27 family and is highly expressed in endothelial cells, where they are involved in angiogenesis, and are also present in the central nervous system, controlling apoptosis [6, 21]. In the present study, miR-27a is up-regulated in the intestinal tissue of sheep with MHC-CE resistance infection with *E. granulosus*, when compared with sheep without this haplotype, suggesting that miR-27a might play an important role in anti-*E. granulosus* infection. Up-regulation of the miR-27 family is also observed in Apicomplexan parasite infection; miR-27a is induced in ECM [10] and miR-27b is associated with TLR4-mediated epithelial antimicrobial defense [29] and apoptosis [4].

It is concluded that these microRNAs, related to the inflammation process, especially the NF-kB pathway, may play an important role in fighting *E. granulosus* infection. However, further studies need to be carried out to verify its function.

## Supplementary Table

**Supplementary Table:** Differential expression levels of miRNAs between CE-R and CE-NR libraries

mi-name	CE-sth	CENR-sth	Fold change	*P* value	Sig-lable
bta-miR-124a	3.21	17.15	2.42	0.00	**
bta-miR-124b	3.21	17.15	2.42	0.00	**
bta-miR-1307	56.33	121.36	1.11	0.00	**
bta-miR-130a	54.55	112.61	1.05	0.00	**
bta-miR-139	31.34	81.11	1.37	0.00	**
bta-miR-145	1827.12	4082.36	1.16	0.00	**
bta-miR-153	0.20	1.25	2.61	0.00	**
bta-miR-16a	46.29	94.28	1.03	0.00	**
bta-miR-16b	43.42	91.26	1.07	0.00	**
bta-miR-181b	334.00	734.81	1.14	0.00	**
bta-miR-181c	11.06	25.39	1.20	0.00	**
bta-miR-181d	62.54	139.47	1.16	0.00	**
bta-miR-18a	2.66	9.49	1.83	0.00	**
bta-miR-193a-3p	8.81	50.27	2.51	0.00	**
bta-miR-193a-5p	30.72	67.64	1.14	0.00	**
bta-miR-196b	18.37	36.87	1.01	0.00	**
bta-miR-202	0.41	1.18	1.52	0.02	*
bta-miR-20b	4.85	11.33	1.23	0.00	**
bta-miR-21-3p	1.57	16.27	3.37	0.00	**
bta-miR-223	57.62	121.66	1.08	0.00	**
bta-miR-22-5p	22.53	51.96	1.21	0.00	**
bta-miR-2284h-5p	0.68	1.69	1.31	0.01	*
bta-miR-2284k	0.27	1.99	2.86	0.00	**
bta-miR-2331-3p	0.27	1.77	2.69	0.00	**
bta-miR-2332	0.89	2.58	1.54	0.00	**
bta-miR-2384	0.27	1.18	2.11	0.00	**
bta-miR-296-5p	2.12	5.45	1.36	0.00	**
bta-miR-29c	472.06	1192.00	1.34	0.00	**
bta-miR-324	3.55	12.95	1.87	0.00	**
bta-miR-331-3p	7.99	66.68	3.06	0.00	**
bta-miR-339a	22.53	91.04	2.01	0.00	**
bta-miR-339b	0.96	4.20	2.13	0.00	**
bta-miR-33a	21.71	124.75	2.52	0.00	**
bta-miR-345-3p	16.86	60.35	1.84	0.00	**
bta-miR-345-5p	8.53	30.03	1.81	0.00	**
bta-miR-34a	2.12	6.99	1.72	0.00	**
bta-miR-365-3p	33.32	71.91	1.11	0.00	**
bta-miR-365-5p	1.50	3.83	1.35	0.00	**
bta-miR-375	1014.30	2087.55	1.04	0.00	**
bta-miR-378b	1.71	14.72	3.11	0.00	**
bta-miR-378c	14.68	166.11	3.50	0.00	**
bta-miR-424-5p	21.30	47.91	1.17	0.00	**
bta-miR-449a	1.16	3.02	1.38	0.00	**
bta-miR-455-3p	27.58	57.55	1.06	0.00	**
bta-miR-490	5.12	11.70	1.19	0.00	**
bta-miR-532	18.16	42.91	1.24	0.00	**
bta-miR-542-5p	0.01	1.18	6.88	0.00	**
bta-miR-551b	0.48	1.40	1.55	0.01	*
bta-miR-582	0.48	1.47	1.62	0.01	**
bta-miR-6123	0.82	3.24	1.98	0.00	**
bta-miR-652	9.15	20.31	1.15	0.00	**
bta-miR-671	0.07	2.65	5.28	0.00	**
bta-miR-708	45.40	108.12	1.25	0.00	**
bta-miR-874	0.96	5.96	2.64	0.00	**
bta-miR-877	5.05	10.16	1.01	0.00	**
bta-miR-99a-3p	4.16	9.35	1.17	0.00	**
oar-miR-106a	3.21	8.61	1.42	0.00	**
oar-miR-10a	1912.32	4800.10	1.33	0.00	**
oar-miR-127	46.70	93.54	1.00	0.00	**
oar-miR-134-5p	0.14	1.84	3.75	0.00	**
oar-miR-154a-3p	1.09	3.09	1.50	0.00	**
oar-miR-16b	445.16	1302.77	1.55	0.00	**
oar-miR-181a	939.13	2073.34	1.14	0.00	**
oar-miR-27a	29.50	273.12	3.21	0.00	**
oar-miR-299-5p	1.37	2.80	1.03	0.01	**
oar-miR-30a-3p	45.20	91.19	1.01	0.00	**
oar-miR-376b-3p	3.62	12.44	1.78	0.00	**
oar-miR-376c-3p	4.57	13.17	1.53	0.00	**
oar-miR-376e-3p	4.92	13.91	1.50	0.00	**
oar-miR-487b-3p	12.77	33.86	1.41	0.00	**
oar-miR-494-3p	10.65	24.14	1.18	0.00	**
oar-miR-495-3p	16.11	38.86	1.27	0.00	**
oar-miR-539-3p	1.09	2.28	1.06	0.01	*
oar-miR-665-3p	0.27	1.18	2.11	0.00	**
bta-miR-1247-5p	0.55	1.40	1.36	0.02	*
bta-miR-216b	2.39	0.81	-1.56	0.00	**
bta-miR-217	1.64	0.44	-1.89	0.00	**
bta-miR-2284m	1.02	0.07	-3.80	0.00	**
bta-miR-2331-5p	5.33	2.50	-1.09	0.00	**
bta-miR-3141	1.84	0.88	-1.06	0.03	*
bta-miR-328	2.73	1.32	-1.04	0.01	**
bta-miR-3432	21.37	10.67	-1.00	0.00	**
bta-miR-484	13.52	6.18	-1.13	0.00	**

Note: CE-R represented the normalized expression level of miRNAs in the small intestine RNA library generated from CE sheep infected with *E. granulosus* infection.

CE-NR represented the normalized expression level of miRNAs in the small intestine RNA library generated from CE-NR sheep with *E. granulosus* infection.

Fold (R/P) and log2 (R/P) indicated the fold change of the miRNAs between samples. *P*-Value manifested the significance of miRNAs differential expression between two samples (*: *P* < 0.05; **: *P* < 0.01).
